# EEG microstates and dynamic functional connectivity reveal stage-specific brain networks in subjective tinnitus

**DOI:** 10.1016/j.isci.2026.116995

**Published:** 2026-07-30

**Authors:** Meng-Fang Gong, Sheng-Yu Tao, Bin Dai, Zhong-Ling Ding, Qian He, Ya-Kang Dai, Ji-Sheng Liu, Duo-Duo Tao

**Affiliations:** 1 Department of Ear, Nose, and Throat, First Affiliated Hospital of Soochow University, Suzhou 215006, China; 2 Kunming Medical University Haiyuan College, Kunming 650106, China; 3 Suzhou Institute of Biomedical Engineering and Technology, Chinese Academy of Sciences, 88 Keling Road, Suzhou 215163, China; 4 University of Science and Technology of China, 96#, Jinzhai Road, Baohe District, Hefei, Anhui 230000, China

**Keywords:** tinnitus, EEG, microstates, dynamic functional network, neural oscillations

## Abstract

Tinnitus with normal hearing suggests central mechanisms, yet stage-specific brain network dynamics remain unclear. This study investigated stage-specific brain network patterns in normal-hearing tinnitus patients using electroencephalography (EEG) microstate and dynamic functional network (DFN) analyses. Resting-state EEG was recorded from 45 participants (15 acute tinnitus, <6 months; 15 chronic tinnitus, ≥6 months; and 15 healthy controls). Results showed that acute tinnitus involved enhanced salience network engagement and reduced central executive network participation, with transitions skewed toward salience processing. Chronic tinnitus exhibited normalized transitions but increased global explained variance, indicating greater network stability. DFN analysis revealed elevated γ-band efficiency in the executive network during acute tinnitus, while chronic tinnitus showed increased low-frequency (δ/β) efficiency in executive and auditory networks. These findings demonstrate distinct neurophysiological profiles across tinnitus stages—acute salience-executive imbalance with aberrant high-frequency synchrony versus chronic compensatory rebalancing through low-frequency adaptation—providing a framework for stage-specific biomarker identification.

## Highlights

• Acute tinnitus reveals salience-executive network imbalance
• Chronic tinnitus has compensatory rebalancing via low-frequency network adaptation
• An integrated microstate and dynamic functional network approach was applied
• All patients had tinnitus with normal hearing, isolating tinnitus-specific effects

## Graphical abstract

**Figure:**
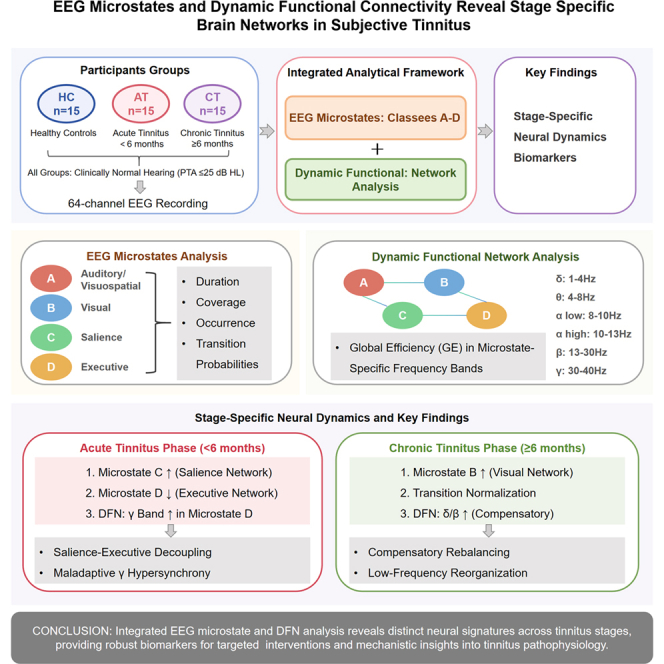

## Teaser

Health sciences; Medicine; Medical specialty; Otorhinolaryngology; Natural sciences; Biological sciences; Neuroscience; Sensory neuroscience; Cognitive neuroscience

## Introduction

Tinnitus, defined as the perception of sound in the absence of an external acoustic stimulus, is a prevalent and distressing neurological disorder that affects approximately 10%–15% of the adult population worldwide.^1^ While tinnitus is frequently associated with hearing loss, a substantial proportion of individuals with clinically normal hearing thresholds (pure-tone average [PTA] ≤ 25 dB HL at 0.5–4 kHz) also experience persistent tinnitus.^2^^,^^3^ In such cases, the peripheral trigger may involve subclinical pathologies not captured by standard audiometry—most notably cochlear synaptopathy (the loss of inner hair cell ribbon synapses) or high-frequency hearing loss above 8 kHz—which in turn initiate central maladaptive plasticity and network-level dysregulation,^4^^,^^5^^,^^6^^,^^7^ operationalized as functional reconfiguration of large-scale brain networks (including the salience, executive, and auditory networks) driven by reduced thalamocortical sensory input, rather than age-related structural atrophy or the direct consequence of threshold hearing loss. The neurophysiological model of tinnitus posits that aberrant neural activity is interpreted as a salient signal that subsequently engages the limbic and executive networks, leading to distress and attentional capture.^1^^,^^8^^,^^9^ A cornerstone theoretical framework for understanding tinnitus neurophysiology is the thalamocortical dysrhythmia (TCD) model.^10^^,^^11^^,^^12^ The TCD model proposes that peripheral auditory deafferentation—whether overt (e.g., high-frequency hearing loss) or subclinical (e.g., cochlear synaptopathy)—leads to reduced sensory input to the thalamus, causing thalamocortical relay neurons to shift from tonic to burst-firing mode, leading to a reduction in normal alpha oscillations and the emergence of pathological cross-frequency coupling, particularly theta-gamma coupling, in the auditory cortex and connected limbic and frontal regions.^12^ This process is hypothesized to involve a large-scale network imbalance, particularly a dysregulation between the salience network (SN), which directs attention to significant stimuli, and the central executive network (CEN), which is responsible for cognitive control and habituation.^13^^,^^14^^,^^15^^,^^16^ Understanding the dynamic interplay between these networks across the progression of tinnitus is crucial for elucidating its pathophysiology.

Electroencephalography (EEG) microstate analysis has emerged as a powerful tool to investigate the rapid spatiotemporal dynamics of large-scale brain networks.^17^ EEG microstates are quasi-stable topographical maps of neuronal activity that persist for tens to hundreds of milliseconds, reflecting the transient synchronization of the underlying neural generators.^18^^,^^19^ Four canonical microstate classes (A–D) have been consistently linked to the activity of distinct resting-state networks: auditory/visuospatial (A), visual (B), salience (C), and central executive (D) networks.^15^^,^^20^ Previous studies applying microstate analysis to tinnitus have primarily focused on patients with chronic tinnitus (CT), revealing alterations in microstate temporal dynamics, such as duration, occurrence, and transition, compared with those in healthy controls (HCs).^13^^,^^16^^,^^21^^,^^22^ However, a significant limitation of these studies is the frequent confounding effect of concomitant hearing loss, making it difficult to distinguish tinnitus-specific neural adaptations from those related to auditory deafferentation.^17^^,^^23^

While microstate analysis excels at characterizing macroscopic temporal dynamics, it offers limited insight into the frequency-specific functional integration within and between these networks. Dynamic functional network (DFN) analysis addresses this gap by quantifying time-varying changes in functional connectivity within specific oscillatory bands (e.g., δ, θ, α, β, and γ), thereby capturing rapid shifts in network efficiency and integration.^24^^,^^25^ DFN approaches have demonstrated sensitivity in capturing state-dependent neural dynamics in other neurological conditions, such as migraine, epilepsy, and chronic pain; however, their application in tinnitus, particularly in relation to microstates, remains nascent.^24^^,^^25^^,^^26^^,^^27^

Importantly, the neural trajectory of tinnitus as it evolves from an acute salient event to a chronic condition likely involves distinct stages of maladaptation and compensation.^23^^,^^28^^,^^29^^,^^30^ However, most previous neuroimaging studies have relied on cross-sectional comparisons between patients with CT and HCs, with few directly comparing patients with acute tinnitus (AT), CT, and well-matched HCs while controlling for the hearing status.^22^^,^^24^^,^^31^ This gap has limited our understanding of the stage-specific neural signatures and dynamic evolution of tinnitus-related brain network changes.

**Figure 1:**
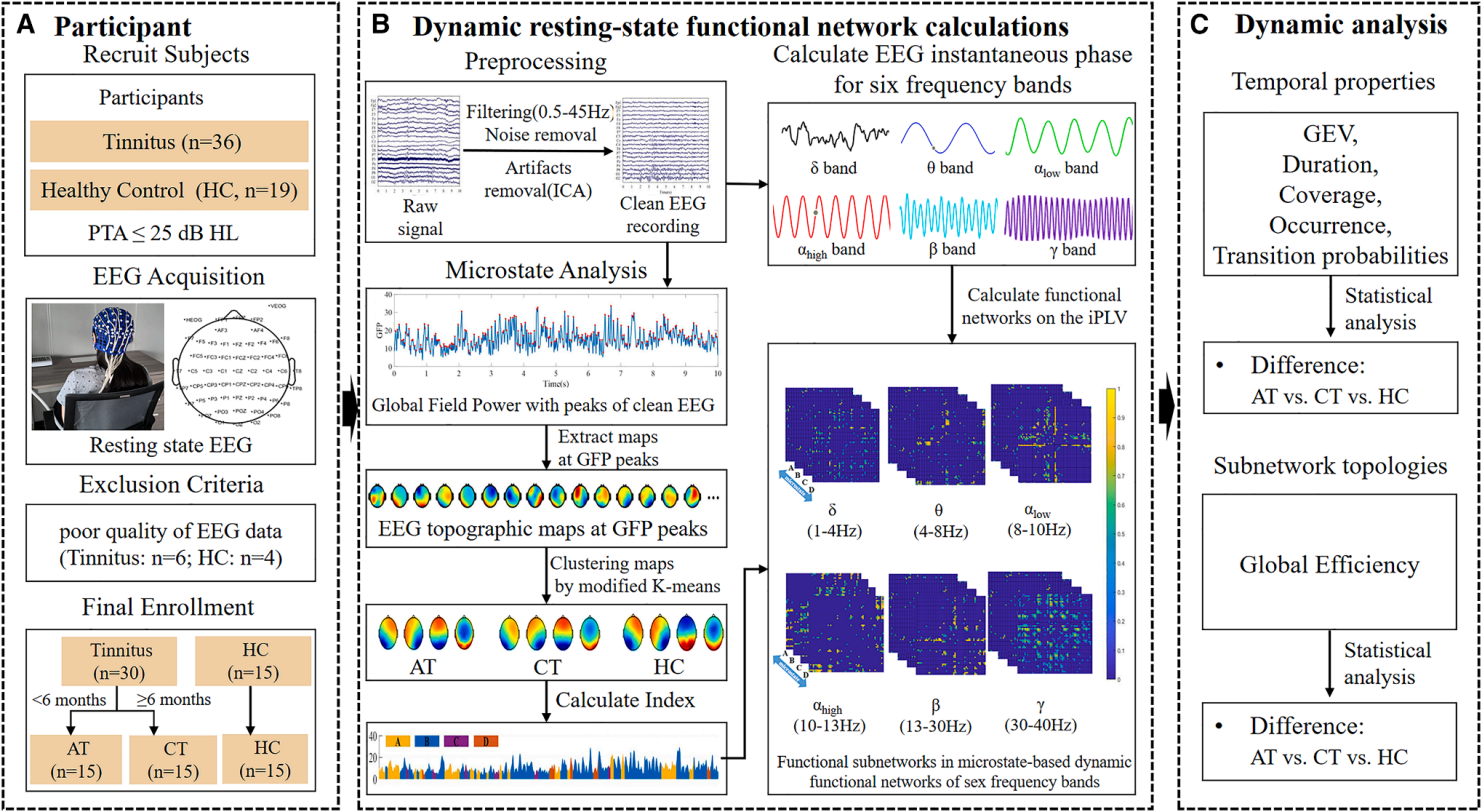
Schematic overview of the experimental workflow Resting-state EEG was recorded in participants grouped by the tinnitus stage (acute tinnitus, AT; chronic tinnitus, CT) and matched healthy controls (HCs). (A) The preprocessing pipeline included signal filtering, re-referencing, and artifact removal. (B) Dynamic resting-state functional networks were derived by segmenting the EEG based on microstate classes (A–D) that were identified using global field power (GFP) peaks and a modified K-means clustering algorithm. The instantaneous phase was then computed across six frequency bands (δ, θ, α-low, α-high, β, and γ) to construct functional networks using the imaginary phase-locking value (iPLV). (C) Data analysis involved comparing the temporal properties of microstates (e.g., GEV, duration, coverage, occurrence, and transition probabilities) and topological properties of the subnetworks (e.g., global efficiency) between the AT, CT, and HC groups. Abbreviations: EEG, electroencephalogram; GEV, global explained variance.

To address these limitations, based on the TCD model and prior neuroimaging evidence, we hypothesized that AT would be characterized by enhanced SN engagement—reflecting the impact of pathological thalamocortical cross-frequency coupling on the insula and the anterior cingulate nodes—and aberrant high-frequency oscillatory activity, reflecting the initial maladaptive response to phantom sound perception; CT would demonstrate compensatory network rebalancing with increased low-frequency oscillatory efficiency, indicative of neuroplastic adaptation; and these stage-specific alterations would be undetectable by conventional static functional connectivity analysis, highlighting the necessity of dynamic approaches. This study introduces an integrative analytical framework that combines EEG microstate analysis with DFN analysis. This combined approach enables a comprehensive investigation that bridges the macroscopic spatiotemporal dynamics and microscopic frequency-specific network efficiency. A key strength of our study design is the rigorous control for hearing loss. All enrolled patients with both acute and CT exhibited clinical normal hearing thresholds (PTA ≤ 25 dB HL at 0.5–4 kHz), thereby minimizing the confounding effect of clinically overt hearing loss on the observed neural changes. We recruited three carefully matched groups: patients with AT, patients with CT, and HCs with normal hearing, to delineate the stage-specific neural signatures and advance the search for objective neurophysiological markers of tinnitus. The experimental workflow is illustrated in Figure 1.

## Results

### Demographic and clinical characteristics

**Figure 2:**
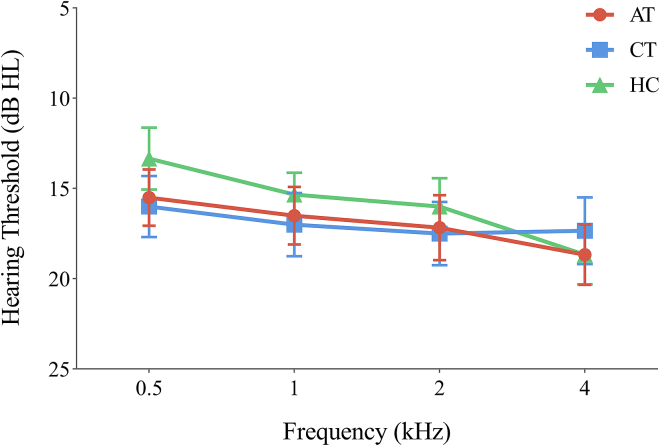
Mean pure-tone audiograms by group Mean pure-tone audiograms (±SEM) for the AT, CT, and HC groups across 0.5, 1, 2, and 4 kHz for both ears combined (*n* = 15 per group).

Table 1 shows a comparison of the demographic data between the three groups. Statistical analysis revealed no significant differences between the AT (*n* = 15), CT (*n* = 15), and HC (*n* = 15) groups in terms of age (*F* = 0.343, *p* = 0.711), sex distribution (χ^2^ = 2.312, *p* = 0.315), and PTA for the tinnitus ear (*t* = 0.287, *p* = 0.776) or for bilateral ears (*F* = 0.166, *p* = 0.847). Subjective tinnitus severity, measured using the Visual Analog Scale (VAS) and Tinnitus Handicap Inventory (THI), showed comparable scores between the AT and CT groups (VAS: AT = 5 [4, 5], CT = 5.4 ± 2.0; THI total: AT = 39.1 ± 16.7, CT = 33.6 ± 18.4; *p* > 0.05, Mann-Whitney U test). Group-averaged audiograms for the three subsamples are presented in Figure 2, confirming matched hearing thresholds across frequencies.

**Table 1:** Demographic and clinical characteristics of the study participants

	AT (*n* = 15)	CT (*n* = 15)	HC (*n* = 15)	Statistic	*p*
Sex (male/female)	9/6	8/7	5/10	*χ*^*2*^ = 2.312	0.315
Age (years)	39.9 ± 9.0	43.3 ± 9.3	42.3 ± 14.8	*F* = 0.343	0.711
PTA (bilateral ears; dB HL)	17.0 ± 5.9	17.0 ± 6.5	15.9 ± 5.2	*F* = 0.176	0.839
PTA (tinnitus ear; dB HL)	17.9 ± 6.2	17.2 ± 6.6	NA	*t* = 0.287	0.776
Duration (months)	4 (2, 5)	28 (12, 60)	NA	*Z* = −4.686	<0.001∗∗∗
Tinnitus ear (L/R/B)	4/7/4	2/3/10	NA	*χ*^*2*^ = 4.838	0.089
VAS	5 (4, 5)	5.4 ± 2.0	NA	*Z* = −0.849	0.412
THI total	39.1 ± 16.7	33.6 ± 18.4	NA	*t* = 0.852	0.402
THI-F	15.1 ± 6.9	12.1 ± 7.6	NA	*t* = 1.110	0.276
THI-E	14.0 ± 7.9	11.5 ± 7.4	NA	*t* = 0.905	0.373
THI-C	10.0 ± 3.9	9.7 ± 6.0	NA	*t* = 0.144	0.887

Data with a normal distribution are presented as mean ± standard deviation, and data with a non-normal distribution are presented as medians (first quartile and third quartile) [M(Q1, Q3)]. Abbreviations: AT, acute tinnitus; CT, chronic tinnitus; HC, healthy control; PTA, pure-tone average; L, left; R, right; B, bilateral; NA, not available; VAS, Visual Analog Scale; THI, Tinnitus Handicap Inventory; THI-F, functional scores on the THI scale; THI-E, emotional scores on the THI scale; THI-C, catastrophic scores on the THI scale. χ^2^, chi-square test; F, one-way analysis of variance (ANOVA). ∗∗∗*p* < 0.001.

### Microstate topographies and temporal dynamics

**Figure 3:**
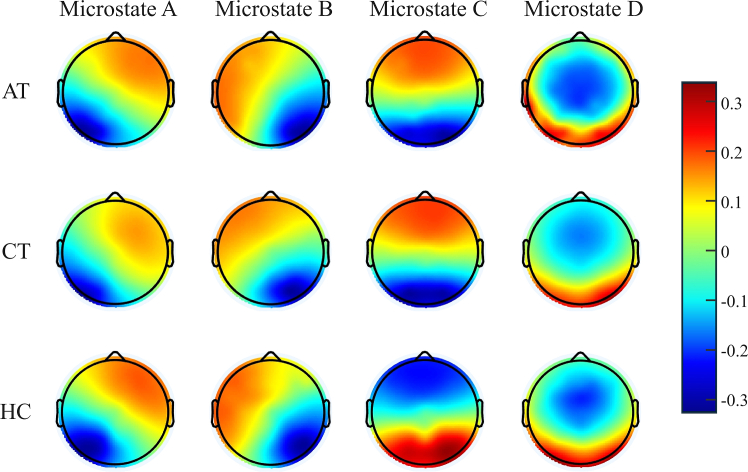
Topographic maps of the four canonical microstate classes The group-level microstate templates (classes A, B, C, and D) are displayed for the acute tinnitus (AT), chronic tinnitus (CT), and healthy control (HC) groups. The topographies are consistent with established neural networks: auditory/visuospatial (A), visual (B), salience (C), and central executive (D).

Cluster analysis identified four canonical microstate classes (A–D) across all participants (Figure 3), with class topographies aligning with established large-scale networks^19^^,^^21^^,^^32^: class A (right anterior-left posterior polarity: auditory/visuospatial processing), class B (left anterior-right posterior: visual network), class C (anterior-posterior orientation: SN), and class D (fronto-central extreme location: CEN). Global explained variance (GEV; the proportion of total EEG variance explained by the four canonical microstate templates, expressed as a percentage) differed significantly across groups (*p* < 0.001). GEV in the CT (74.3%) group was significantly higher than that in the AT (69.5%, *p* = 0.002, d = 1.129) and HC (65.7%, *p* < 0.001, d = 2.144) groups, while GEV in the AT group was significantly higher than that in the HC group (*p* = 0.007, d = 0.960) (Table 2).

**Table 2:** Comparison of microstate parameters between the AT, CT, and HC groups

	AT (*n* = 15)	CT (*n* = 15)	HC (*n* = 15)	*p* value	AT vs. CT		AT vs. HC		CT vs. HC	
					*p* value	Cohen’s d	*p* value	Cohen’s d	*p* value	Cohen’s d
GEV (%)	69.5 ± 4.2	74.3 ± 4.3	65.7 ± 3.7	<0.001	0.002	−1.129	0.007	0.960	<0.001	2.144

**Microstate duration (ms)**										

A	77.4 ± 12.8	77.3 ± 12.5	73.3 ± 11.1	0.421	NA	NA	NA	NA	NA	NA
B	73.2 ± 10.9	82.6 ± 22.2	68.1 ± 7.4	**0.034∗**	0.603	NA	0.560	NA	**0.028∗**	0.873
C	95.6 ± 20.8	89.2 ± 19.1	81.5 ± 15.6	0.057	NA	NA	NA	NA	NA	NA
D	73.9 ± 14.0	91.6 ± 27.3	84.6 ± 11.8	**0.038∗**	0.073	NA	0.090	NA	1	NA

**Microstate occurrence (times/s)**										

A	2.9 ± 0.8	2.5 ± 0.8	3.0 ± 0.6	0.130	NA	NA	NA	NA	NA	NA
B	2.9 ± 0.5	2.8 ± 0.7	2.6 ± 0.5	0.242	NA	NA	NA	NA	NA	NA
C	3.7 ± 0.6	3.0 ± 0.5	3.5 ± 0.4	**0.003∗∗**	**<0.001∗∗∗**	1.190	0.310	NA	**0.016∗**	−0.986
D	2.8 ± 0.6	3.1 ± 0.7	3.6 ± 0.8	**0.005∗∗**	0.728	NA	**0.005∗∗**	−1.149	0.150	NA

**Microstate coverage (%)**										

A	22.9 ± 7.7	20.0 ± 8.0	22.2 ± 5.6	0.516	NA	NA	NA	NA	NA	NA
B	21.4 ± 4.3	23.4 ± 10.0	18.2 ± 4.9	0.111	NA	NA	NA	NA	NA	NA
C	35.2 ± 8.4	27.7 ± 8.8	28.5 ± 6.2	**0.025∗**	**0.013∗**	0.872	**0.026∗**	0.908	0.781	NA
D	20.6 ± 4.4	28.9 ± 12.0	31.0 ± 9.4	**0.005∗∗**	0.063	NA	**0.005∗∗**	−1.417	1	NA

Intergroup comparisons were performed using one-way ANOVA, and pairwise comparisons were conducted using the Bonferroni post hoc test. Cohen’s d was used to quantify the effect sizes of significant findings. Abbreviations: AT, acute tinnitus; CT, chronic tinnitus; HC, healthy control; GEV, global explained variance. ∗*p* < 0.05, ∗∗*p* < 0.01, ∗∗∗*p* < 0.001.

### Stage-specific alterations in microstate parameters

**Figure 4:**
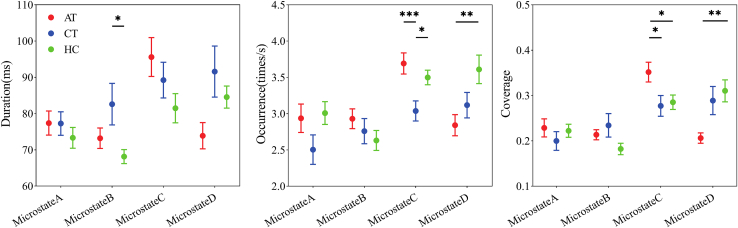
Group differences in microstate temporal parameters Bar graphs (mean ± SEM) illustrate the group differences in (A) the duration of microstate B, (B) occurrence, and (C) coverage of microstates C and D among the acute tinnitus (AT), chronic tinnitus (CT), and healthy control (HC) groups. ∗*p* < 0.05, ∗∗*p* < 0.01, and ∗∗∗*p* < 0.001; post hoc Bonferroni test.

The different tinnitus stages exhibited distinct microstate patterns (Figure 4 and Table 2). The three groups demonstrated significant differences across multiple microstate parameters, specifically concerning the duration of microstate B (F[2,42] = 3.623, *p* = 0.035) and the occurrence and coverage of microstates C (occurrence: F[2,42] = 6.722, *p* = 0.003; coverage: F[2,42] = 4.025, *p* = 0.025) and D (occurrence: F[2,42] = 5.040, *p* = 0.011; coverage: F[2,42] = 5.389, *p* = 0.008). Post hoc tests revealed that the CT group displayed prolonged duration of microstate B compared with that in the HC group (82.6 vs. 68.1 ms; *p* = 0.028, d = 0.873, corrected) and a non-significant trend toward a longer microstate D than that in the AT group (91.6 vs. 73.9 ms; *p* = 0.073, uncorrected). AT showed elevated microstate C occurrence compared with that in the CT group (3.7 vs. 3.0 times/s; *p* < 0.001, d = 1.190), alongside reduced microstate D occurrence versus the HC group (2.8 vs. 3.6; *p* = 0.005, d = −1.149, corrected). The CT group showed decreased microstate C occurrence compared with that in the HC group (3.0 vs. 3.5 times/s; *p =* 0.016, d = −0.986, corrected). AT demonstrated broader microstate C coverage than that in both the CT (35.2% vs. 27.7%; *p* = 0.013, d = 0.872, corrected) and HC groups (35.2% vs. 28.5%; *p* = 0.026, d = 0.908, corrected), whereas microstate D coverage was lower in the AT group than in the HC group (20.6% vs. 31.0%; *p* = 0.005, d = −1.417, corrected).

### Network transition reconfiguration

**Figure 5:**
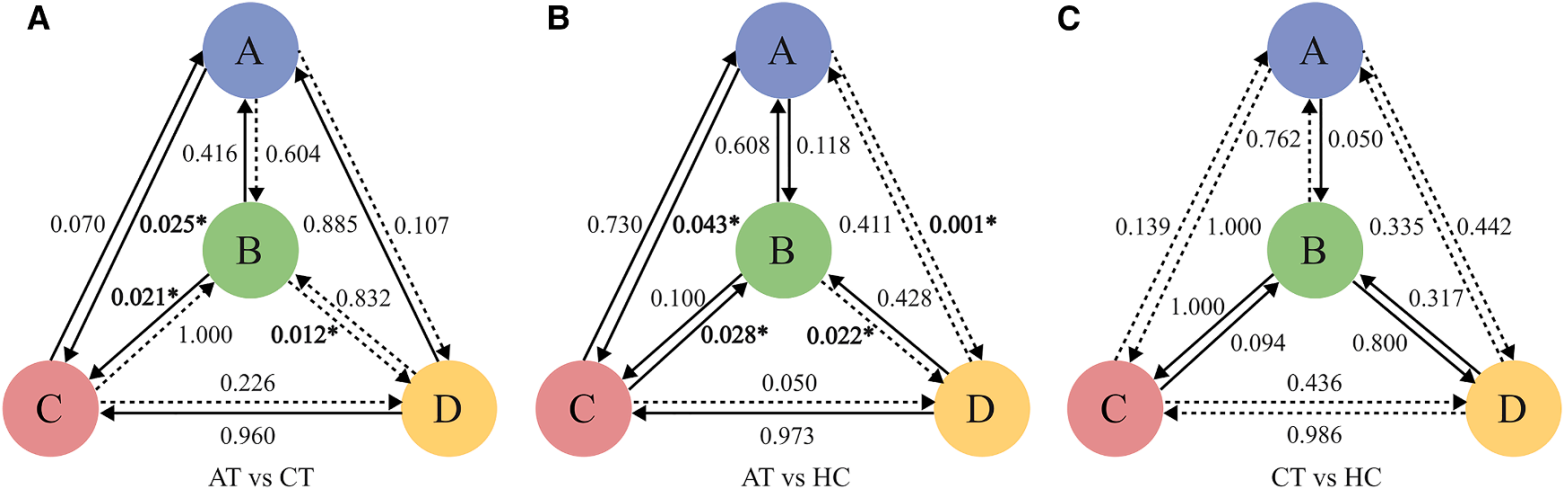
Altered transition probabilities between the microstate classes The directed graphs show significant differences in transition probabilities between the four microstate classes (A, B, C, and D) in the acute tinnitus (AT), chronic tinnitus (CT), and healthy control (HC) groups. Solid arrows indicate a significantly higher probability of transition in the former group than in the latter, whereas dashed arrows denote a significantly lower probability. ∗*p* < 0.05.

The transition probability (TP) differed markedly among stages (Figure 5). The AT group exhibited heightened shifts toward salience-related processing (A to C: AT = 42.8% vs. HC = 35.7%, *p* = 0.043; AT = 42.8% vs. CT = 33.4%, *p* = 0.025; B to C: AT = 42.0% vs. CT = 34.1%, *p* = 0.021) but impaired shifts to executive networks (B to D: AT = 27.9% vs. CT = 38.8%, *p* = 0.012; AT = 27.9% vs. HC = 37.8%, *p* = 0.022; A to D: AT = 26.5% vs. HC = 38.6%, *p* = 0.001 corrected). In contrast, the CT group exhibited balanced transitions similar to those in the HC group, with microstate B to D probabilities restored to normative levels (CT = 38.8% vs. HC = 37.8%, *p* = 0.800).

### DFN and static functional network

**Figure 6:**
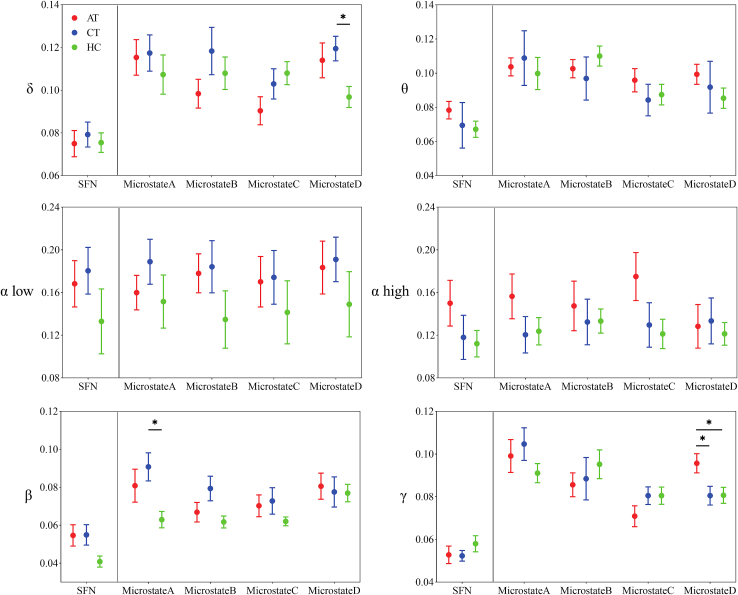
Comparison of global efficiency between static and dynamic functional networks Bar graphs (mean ± SEM) depict the global efficiency for the three groups derived from the SFN analysis and microstate-based DFN analysis across different frequency bands and microstates (A, B, C, and D). Significant group differences identified using DFN analysis are indicated with ∗. Abbreviations: SFN, static functional network; DFN, dynamic functional network. ∗*p* < 0.05.

Global efficiency (GE) represents the integration capacity of the brain network (Figure 6 and Table 3). Table 3 shows the GE results for the three functional network groups. One-way ANOVA showed no significant difference in GE at all frequencies using the conventional static functional network (SFN) method; however, significant differences were observed in GE based on DFN analysis results. The DFN analysis found significant differences across the three groups, primarily manifested in the δ frequency band in microstate D (F[2,42] = 3.391, *p* = 0.043), which was significantly different from both the β frequency band in microstate A (F[2,42] = 4.024, *p* = 0.025) and the γ frequency band in microstate D (F[2,42] = 5.169, *p* = 0.010). Post hoc Bonferroni comparison analyses showed that the efficiency of the δ frequency band in microstate D was significantly higher for the CT (0.1195) group than for the HC group (0.0968, *p* = 0.049); the efficiency of the γ frequency band in microstate D was significantly higher for the AT (0.0957) group than for the HC (0.0807, *p* = 0.040) or CT (0.0793, *p* = 0.015) groups; the efficiency of the β frequency band in microstate A was significantly higher for the CT group (0.0907) than for the HC group (0.0629, *p* = 0.023). No significant differences were noted in the remaining comparisons (all *p* > 0.05).

**Table 3:** Global efficiency of functional networks between the AT, CT, and HC groups

Bands	Groups	SFN	DFN				Post hoc tests
			Microstate A	Microstate B	Microstate C	Microstate D	
δ	AT	0.0750 ± 0.0238	0.1153 ± 0.0323	0.0983 ± 0.0261	0.0903 ± 0.0255	0.1140 ± 0.0315	**CT > HC**, ***p* = 0.049∗**, Cohen’s d = 1.093
	CT	0.0792 ± 0.0261	0.1174 ± 0.0329	0.1183 ± 0.0429	0.1029 ± 0.0274	0.1195 ± 0.0223	
	HC	0.0754 ± 0.0177	0.1073 ± 0.0355	0.1079 ± 0.0294	0.1080 ± 0.0208	0.0968 ± 0.0191	
	*p* value	0.841	0.688	0.277	0.145	**0.043∗**	
θ	AT	0.0783 ± 0.0198	0.1037 ± 0.0204	0.1026 ± 0.0207	0.0959 ± 0.0264	0.0993 ± 0.0227	–
	CT	0.0695 ± 0.0518	0.1088 ± 0.0620	0.0969 ± 0.0489	0.0843 ± 0.0358	0.0918 ± 0.0588	–
	HC	0.0671 ± 0.0183	0.0998 ± 0.0365	0.1100 ± 0.0227	0.0874 ± 0.0233	0.0854 ± 0.0229	–
	*p* value	0.634	0.848	0.562	0.532	0.616	–
α-low	AT	0.1682 ± 0.0839	0.1599 ± 0.0627	0.1780 ± 0.0706	0.1701 ± 0.0915	0.1834 ± 0.0961	–
	CT	0.1804 ± 0.0846	0.1888 ± 0.0814	0.1842 ± 0.0947	0.1742 ± 0.0974	0.1910 ± 0.0808	–
	HC	0.1330 ± 0.1178	0.1515 ± 0.0964	0.1347 ± 0.1042	0.1415 ± 0.1144	0.1490 ± 0.1182	–
	*p* value	0.386	0.426	0.279	0.633	0.475	–
α-high	AT	0.1500 ± 0.0829	0.1564 ± 0.0812	0.1475 ± 0.0899	0.1750 ± 0.0871	0.1283 ± 0.0792	–
	CT	0.1180 ± 0.0801	0.1204 ± 0.0660	0.1323 ± 0.0827	0.1296 ± 0.0809	0.1334 ± 0.0833	–
	HC	0.1121 ± 0.0480	0.1237 ± 0.0497	0.1333 ± 0.0438	0.1212 ± 0.0531	0.1213 ± 0.0411	–
	*p* value	0.312	0.276	0.826	0.121	0.894	–
β	AT	0.0546 ± 0.0217	0.0808 ± 0.0336	0.0669 ± 0.0199	0.0702 ± 0.0223	0.0805 ± 0.0267	**CT > HC**, ***p* = 0.023∗**, Cohen’s d = 1.187
	CT	0.0549 ± 0.0209	0.0907 ± 0.0286	0.0793 ± 0.0251	0.0728 ± 0.0270	0.0775 ± 0.0308	
	HC	0.0408 ± 0.0114	0.0629 ± 0.0167	0.0617 ± 0.0122	0.0620 ± 0.0090	0.0769 ± 0.0178	
	*p* value	0.071	**0.025**∗	0.053	0.345	0.918	
γ	AT	0.0528 ± 0.0159	0.0991 ± 0.0299	0.0856 ± 0.0217	0.0709 ± 0.0189	0.0957 ± 0.0173	**AT > HC**, ***p* = 0.040∗**, Cohen’s d = 0.940; **AT > CT**, ***p* = 0.015∗**, Cohen’s d = 0.962
	CT	0.0523 ± 0.0097	0.1047 ± 0.0296	0.0884 ± 0.0385	0.0785 ± 0.0157	0.0793 ± 0.0168	
	HC	0.0580 ± 0.0146	0.0910 ± 0.0173	0.0952 ± 0.0261	0.0805 ± 0.0157	0.0807 ± 0.0145	
	*p* value	0.458	0.370	0.662	0.213	**0.010∗**	

Intergroup comparisons were performed using one-way ANOVA, and pairwise comparisons were conducted using the Bonferroni post hoc test. Cohen’s d was used to quantify the effect sizes of significant findings. Abbreviations: SFN, static functional network; DFN, dynamic functional network; AT, acute tinnitus; CT, chronic tinnitus; HC, healthy control. ∗*p* < 0.05.

**Figure 7:**
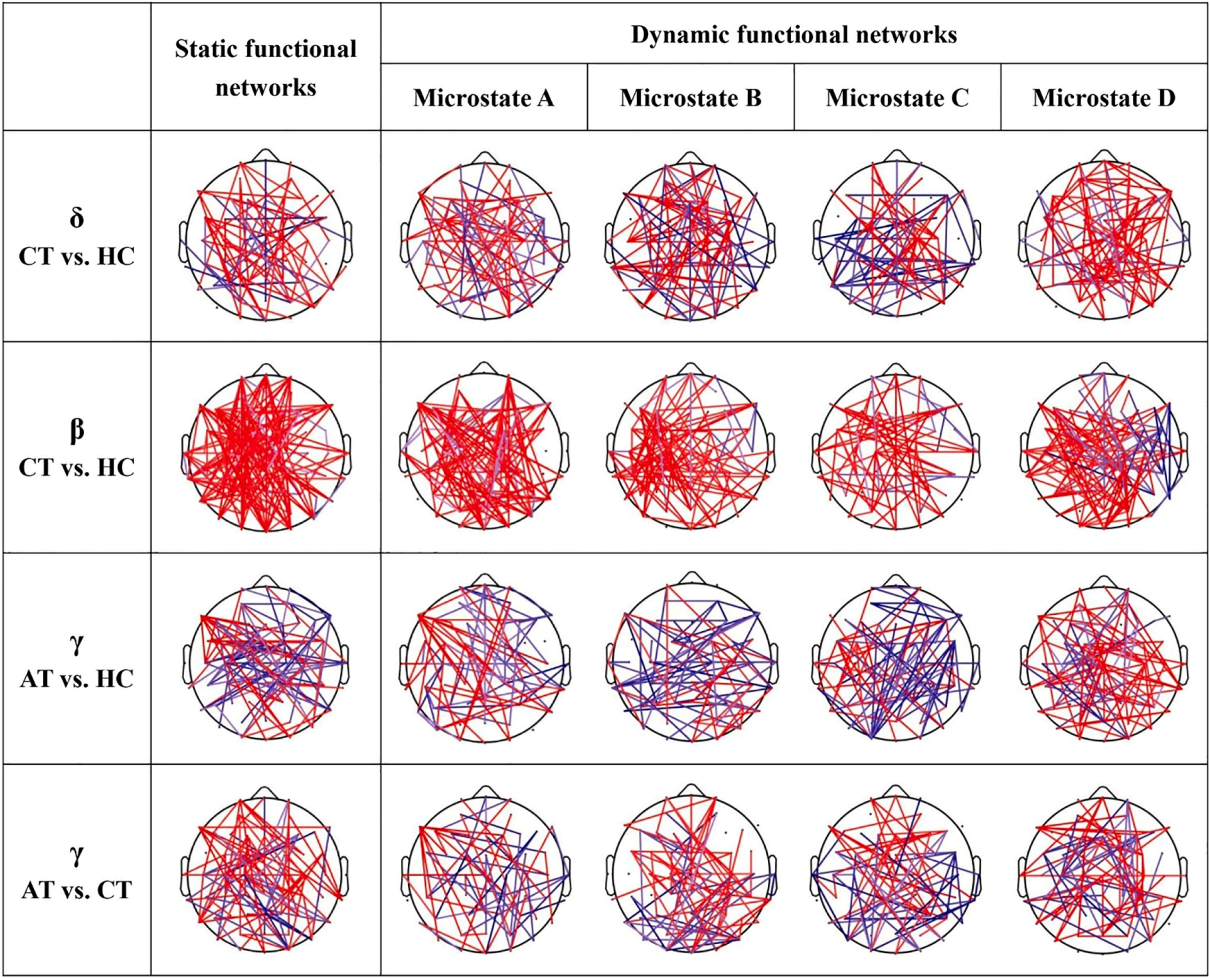
Contrasts in functional connectivity patterns between groups The matrices compare the average imaginary phase-locking value (iPLV) connectivity for significant frequency bands and microstates. The first column represents the traditional SFNs. Columns 2–5 represent the DFNs for each microstate class (A, B, C, and D). The red lines denote stronger synchronization in the former group (labeled) than in the latter, whereas the blue lines denote weaker synchronization. Abbreviations: SFN, static functional network; DFN, dynamic functional network.

These DFN differences were visualized by contrasting the averaged imaginary phase-locking value (iPLV) connectivity matrices between the groups for the significant frequency bands (Figure 7). The analysis revealed that the group differences were not uniformly distributed across the brain states but were specifically linked to particular microstates. For instance, in the δ band, the CT group exhibited a pattern of elevated synchronization compared with that in the HC group, which was particularly pronounced during microstate D (CEN). Conversely, in the γ band, the AT group demonstrated significantly stronger functional connectivity than that in both the HC and CT groups, specifically during microstate D. This γ-band hypersynchronization in the AT group was not apparent in the SFN analysis, which only showed an overall weaker synchronization trend. Similarly, the enhanced β band efficiency in CT during microstate A (auditory/visuospatial network) was reflected in more robust functional connections compared with that in HCs.

## Discussion

This study provides the first systematic evidence of spatiotemporal and frequency-specific alterations in global neural dynamics in patients with tinnitus with normal hearing by integrating EEG microstate analysis with DFN analysis. These findings reveal stage-specific differences in brain network dynamics between acute and CT. AT was characterized by SN hyperactivation and gamma-band executive network hypersynchronization, whereas CT showed restored transition probabilities and increased low-frequency (δ/β) efficiency. This non-monotonic pattern indicates active neuroplastic adaptation rather than uniform progressive deterioration.

### Stage-specific reconfiguration of network dynamics: From salience-driven imbalance to compensatory rebalancing

#### Microstate topographies and temporal dynamics

The topographies of the four canonical microstate classes (A–D) demonstrated strong consistency with the findings of previous research,^19^^,^^21^^,^^32^ mapping to the auditory/visuospatial (A), visual (B), salience (C), and central executive (D) networks.^15^ A key finding was the significantly elevated GEV in patients with CT (GEV = 74.3%), which exceeded that of both HCs (65.7%, *p* < 0.001) and acute-phase patients (69.5%, *p* = 0.002), with clinically significant effect sizes (Cohen’s d > 0.96). As the GEV quantifies the extent to which brain activity is explained by a limited set of patterns, higher values indicate more stereotyped or rigid dynamics.^33^ The observed GEV gradient (HC < AT < CT) suggests increased spatiotemporal regularity across stages. The intermediate GEV in patients with AT may reflect early compensatory mechanisms triggered by auditory deprivation, whereas the surge in patients with CT indicates the stabilization of maladaptive patterns, aligning with tinnitus centralization processes,^7^^,^^8^^,^^30^ which was strongly supported by the large effect size (d = 2.144).

#### AT: A critical window of salience-executive decoupling

Patients with AT exhibited alterations in microstate dynamics that are commonly associated with the SN and CEN. Specifically, we observed an increase in microstate C (often linked to SN activity) coverage and a decrease in microstate D (often linked to CEN engagement) occurrence, indicating a neurocognitive state dominated by limbic-driven threat detection, which aligns with the findings of studies on early stage chronic pain.^26^^,^^34^ Mechanistically, these stage-specific patterns align with the predictions of the TCD model.^10^^,^^35^ According to this model, reduced α-band inhibition permits pathological θ-γ cross-frequency coupling, which is broadcast to the insula via hyperactive microstate C (SN). In AT, this thalamocortical burst firing abnormally engages executive circuitry, as reflected in the observed gamma-band hypersynchrony within microstate D. Crucially, the observed SN-CEN decoupling (increased A→C, B→C; decreased A→D, B→D transition probabilities) points to failed top-down regulation rather than solely bottom-up signal generation. This distinction explains how unattended salience signals escalate limbic processing without executive modulation, potentially contributing to the distress often associated with early tinnitus.^36^^,^^37^ In CT, the observed shift toward δ/β-mediated efficiency in the executive and auditory networks instead suggests a homeostatic renormalization of thalamocortical oscillatory dynamics, enabling partial network rebalancing.

#### Chronic adaptation: Cross-modal compensation and incomplete network rebalancing

The transition to chronicity was marked by adaptive shifts: (1) cross-modal compensation: prolonged microstate B duration (increased by 21% vs. HC, *p* = 0.028) aligns with increased resting-state connectivity between the visual and dorsolateral prefrontal regions,^38^^,^^39^ suggesting multisensory reorganization to counteract persistent auditory phantom signals; (2) partial CEN restoration: transition probabilities in the CT group began resembling those in HCs (CT = 38.8% vs. AT = 27.9%, *p* = 0.012; CT = 38.8% vs. HC = 37.8%, *p* = 0.800), indicating neural adaptation over time.^21^^,^^22^^,^^40^ This restoration may occur through prefrontal inhibitory mechanisms.^41^ However, persistent alterations, such as prolonged microstate B duration and decreased microstate C occurrence, indicate that the brain establishes a new, relatively stable functional configuration rather than fully returning to its original state.^22^ Comparative studies reinforce that while patients with CT show partial normalization, significant differences in microstate temporal characteristics and syntax persist, unlike the pronounced disruptions observed in AT or idiopathic sudden sensorineural hearing loss (ISSNHL) with tinnitus.^21^^,^^39^

### DFN analysis: Unmasking frequency-specific efficiency alterations

The DFN analysis revealed that network efficiency changes were undetectable using microstate temporal parameters or SFN analysis. While SFN often fails to identify group differences due to insensitivity to transient dynamics, DFN captures rapid, frequency-specific alterations.^42^^,^^43^ Multiple studies have confirmed this sensitivity, with temporal lobe epilepsy-induced reorganization being detectable only in dynamic analyses.^25^

Mechanistically, AT was characterized by a significantly increased GE within the γ band for microstate D (CEN).The γ hypersynchronization may reflect a maladaptive state of executive network overactivity and inefficiency, which impairs regulatory control over the SN.^44^ In contrast, CT exhibited increased GE in lower frequency bands, specifically β for microstate A (auditory/visuospatial) and δ for microstate D. This pattern suggests the compensatory engagement of low-frequency oscillations (β for sensory gating and δ for inhibition) to exert more effective inhibitory control, reducing tinnitus salience,^45^ which is consistent with theories of homeostatic and maladaptive plasticity.^46^

### Integration of microstate and DFN findings: Toward a unified model of tinnitus

Integrating microstate and DFN analyses provides a comprehensive pathophysiological model that captures both macroscale network imbalances and microscale nodal efficiency changes across stages, offering superior mechanistic insights and sensitivity compared to static-or single-modality analyses.^25^^,^^47^

In the acute stage, microstate analysis shows a breakdown in dynamic equilibrium toward SN dominance (increased microstate C frequency/duration).^13^ The DFN analysis showed that this is accompanied by an abnormally high GE in the γ band within the executive network (microstate D), suggesting maladaptive hypersynchronization that traps the brain in a state of persistent vigilance, impairing normal network interactions.^23^ This co-occurrence of network imbalance and aberrant nodal efficiency is supported by findings in related conditions such as cognitive fatigue and epilepsy.^25^^,^^48^ Static methods often miss these rapid changes,^16^ and microstate analysis alone lacks spectral details.^22^

In the chronic stage, microstate analysis indicated the restoration of more balanced transitions and increased stability of microstate D. DFN revealed that this macroscopic equilibrium is achieved through spectral reorganization; enhanced low-frequency (δ/β) activity in executive and sensory networks is associated with effective inhibition and regulation,^49^^,^^50^ representing the adaptation of the brain to establish a new steady state. These findings are corroborated by studies showing widespread network reorganization and decreased flexibility in patients with CT.^24^^,^^44^ This integrated model accounts for both acute maladaptive hypersynchronization and chronic compensatory rebalancing.

Integrating our multimodal findings provides convergent mechanistic insights. Reduced anterior cingulate GABA+/creatine ratios in AT^30^ mechanistically align with the observed SN hyperengagement (increased microstate C) and gamma-band executive network hypersynchrony, consistent with GABAergic disinhibition driving limbic salience attribution. The normalization of both GABA+ levels and microstate transition probabilities in CT suggests a coordinated neurochemical-neurophysiological compensatory process. Complementarily, graph-theoretical fMRI analysis^29^ revealed reduced prefrontal-limbic local efficiency in AT with partial restoration in the chronic stage, directly paralleling our finding of increased low-frequency (δ/β) global efficiency in executive and auditory microstates. Together, these multimodal data support a model wherein AT is characterized by GABAergic deficits, salience hyperactivation, and gamma dysrhythmia, while CT exhibits neurochemical normalization, low-frequency oscillatory adaptation, and partial topological recovery.

### Clinical implications and future directions

Our findings have several important clinical implications. Identifying the acute-stage pattern of “SN hyperactivation coupled with high-frequency executive network inefficiency” could help in identifying high-risk patients, enabling early intervention. Future therapies (e.g., neurofeedback and transcranial magnetic stimulation (TMS)) could be tailored to target specific networks and oscillatory frequencies (e.g., acutely reducing aberrant γ synchrony or promoting beneficial δ activity chronically).^37^^,^^51^^,^^52^

### Data duration and measurement stability

The representativeness of the 30-s data epochs was supported by a test-retest consistency analysis comparing the original measurements with those derived from an independent set of three 10-s segments. Global and proportional parameters (GEV and microstate coverage) showed good-to-excellent consistency (intraclass correlation coefficient (ICC) = 0.884–0.905), indicating that 30-s segments provide stable estimates of the overall microstate landscape. Duration and occurrence parameters exhibited higher between-segment variability (ICC = 0.436–0.478), but the between-subject ranking patterns remained highly stable (Pearson r = 0.465–0.930), which is sufficient for group-level comparisons. Transition probabilities demonstrated moderate-to-good consistency (mean ICC = 0.762), supporting reliable estimation of microstate dynamics. All six frequency bands of the SFN showed good stability (mean r = 0.889 and mean ICC = 0.882), with particularly high consistency in the alpha bands (ICC > 0.92). Across the 24 DFN parameters (6 bands × 4 microstates), overall stability was also good (mean r = 0.770 and mean ICC = 0.764). The alpha bands again showed the highest consistency (ICC > 0.87), whereas the δ, θ, β, and γ bands exhibited moderate-to-good stability (ICC range: 0.68–0.75). These findings confirm that 30 s of artifact-free resting-state EEG data yield reliable and temporally stable estimates of the microstate parameters and network metrics central to this study (Table S1).

In summary, by integrating EEG microstate and DFN analyses, this study delineated the specific spatiotemporal and frequency-specific reorganization of brain networks across tinnitus stages. The acute stage is marked by SN-driven imbalance and aberrant high-frequency synchrony, whereas the chronic stage demonstrates compensatory rebalancing via low-frequency oscillatory reorganization. These findings provide a novel, multidimensional, and dynamic framework for understanding the pathophysiology of tinnitus, thereby laying a solid theoretical foundation for the development of biomarkers and therapeutic strategies based on network dynamics and neural oscillations.

### Limitations of the study

The cross-sectional design limits conclusions regarding temporal dynamics, as the findings reflect between-group differences rather than within-subject longitudinal changes. Future longitudinal studies tracking patients from tinnitus onset to the chronic stage are needed to validate these neural alterations and clarify the progression of tinnitus. In addition, replication in larger independent cohorts is necessary to confirm the generalizability of the findings and support subgroup analyses (e.g., tinnitus laterality and sound characteristics).

Secondly, this study did not follow the recommendations of the TINnitus research NETwork (TINNET) initiative, namely to administer the Tinnitus Resting State Questionnaire (TRSQ) immediately after the completion of EEG recording. Consequently, we were unable to quantify the participants’ level of attention to tinnitus or the activity of spontaneous thoughts during the resting-state recording period. Although real-time monitoring and standardized verbal instructions mitigated state-related variability to some extent, they cannot replace validated self-report scales. The absence of TRSQ data also limits the direct comparability of this study’s findings with datasets that meet TINNET standards. Future studies should incorporate these scales to distinguish stable, trait-like network alterations from state-dependent fluctuations.

Thirdly, as this study did not assess extended high-frequency hearing tests or distortion product otoacoustic emissions (DPOAEs), the potential confounding influence of latent hearing loss or cochlear synaptic lesions on the study results cannot be ruled out. Future research should investigate this unmeasured source of inter-individual variation.

This connectivity analysis has two primary limitations. First, iPLV eliminates volume conduction but discards zero-lag synchronization, potentially underestimating genuine physiological interactions with minimal phase delay. Second, sensor-space analysis precludes precise anatomical localization of network changes. Although microstate classes (A–D) provide functional proxies, they lack the spatial resolution of source modeling. Future studies should employ distributed source reconstruction (e.g., exact low-resolution brain electromagnetic tomography (eLORETA)) to confirm the specific cortical generators underlying the observed stage-specific network alterations.^53^

Although our consistency analysis indicates that 30-s datasets demonstrate good to excellent temporal stability for both microstate and functional connectivity analyses, the absolute consistency regarding duration and occurrence rates is low (ICC < 0.50). Future studies aimed at characterizing finer temporal dynamics or requiring absolute temporal precision may benefit from longer continuous recordings.

## Resource availability

### Lead contact

Requests for further information and resources should be directed to and will be fulfilled by the lead contact, Duo-Duo Tao (entdtao@163.com).

### Materials availability

This study did not generate new materials.

### Data and code availability

• De-identified human EEG data have been deposited at Mendeley Data: https://doi.org/10.17632/npfx2zhb2r.1. They are publicly available as of the date of publication.
• This paper does not report original code.
• Any additional information required to reanalyze the data reported in this paper is available from the lead contact upon request.

## Acknowledgments

We thank the 10.13039/501100001809National Natural Science Foundation of China (grant nos. 82571311 and 82171159), the Jiangsu Provincial Health Commission Scientific Research Project (grant no. K2024079), the 10.13039/501100018556Science and Technology Program of Suzhou (grant no. SKY2023043), the Suzhou Basic Research Pilot Project (grant no. SSD2024025), the Postgraduate Research & Practice Innovation Program of Jiangsu Province (grant no. KYCX24_3346), the AI Innovation Key Projects of SIBET (grant no. E455380101), and the Suzhou Key Laboratory of Artificial Intelligence in Biomedical Engineering (grant no. SZS2024007) for their funding and support. We also thank the members of our research group for their insightful discussions and support.

## Author contributions

Conceptualization, design, methodology, funding acquisition, project administration, resources, and supervision, D.-D.T., J.-S.L., and Y.-K.D.; data curation and formal analysis, M.-F.G., S.-Y.T., B.D., Z.-L.D., and Q.H.; visualization, writing – original draft, and investigation, M.-F.G., S.-Y.T., and B.D.; writing – review and editing, M.-F.G. and D.-D.T.

## Declaration of interests

The authors declare no competing interests.

## STAR★Methods

### Key resources table

**Table:** 

REAGENT or RESOURCE	SOURCE	IDENTIFIER
**Deposited data**		

Raw EEG data	This study	https://doi.org/10.17632/npfx2zhb2r.1

**Software and algorithms**		

MATLAB	MathWorks	https://www.mathworks.com
EEGLAB	NA	http://sccn.ucsd.edu/eeglab/index.html
GraphPad Prism	GraphPad Software, Inc.	https://www.graphpad.com
Curry 8	Compumedics Neuroscan	https://compumedicsneuroscan.com

### Experimental model and study participant details

#### Human participants

A total of 36 outpatients with subjective tinnitus and clinically normal hearing (PTA ≤25 dB HL at 0.5-4 kHz) were initially enrolled, after being rigorously screened for absence of hearing loss. Concurrently, 19 age- and sex-matched healthy individuals without tinnitus were recruited. Rigorous matching protocols were applied to control for educational background and hearing thresholds, thereby minimizing confounding factors. All participants displayed right-handed dominance (Edinburgh Handedness Inventory score ≥70).

This prospective case-control study received ethical approval from the Ethics Committee of the First Affiliated Hospital of Soochow University (IRB No. 2025147) and was conducted in accordance with the Declaration of Helsinki and relevant national regulatory standards, and was conducted at the Department of Otolaryngology, First Affiliated Hospital of Soochow University, from January to March 2025. Written informed consent was obtained prospectively from all participants.

### Method details

#### Experimental workflow

Figure 1 outlines the overall framework to study the microstate in tinnitus. (a) Resting-state EEG data of patients with tinnitus were collected and divided into two groups according to the different stages of tinnitus. (b) Dynamic resting-state functional network calculations. First, raw EEG signal preprocessing included signal filtering, average re-referencing, and artifact removal. Second, the clean EEG recordings were subjected to microstate analysis. GFP peaks were identified, and topographic maps of these peaks were extracted and clustered into representative microstate classes (labeled as AT, CT, and HC in the figure) using a modified K-means algorithm. Subsequently, the EEG instantaneous phase was calculated across six frequency bands: δ, θ, α-low, α-high, β, and γ. Functional networks were constructed based on the iPLVs, resulting in microstate-based dynamic functional subnetworks for each frequency band. (c) Data analysis. The temporal properties of the microstates, including the GE, duration, coverage, occurrence, and transition probabilities, were computed. Additionally, subnetwork topological properties, such as GE, were evaluated. Statistical analyses were conducted to identify the differences in these metrics among the AT, CT, and HC groups.

#### EEG-driven exclusion process

Pre-EEG screening-based exclusion criteria included (1) otologic comorbidities (Meniere's disease, otosclerosis), 2) psychiatric diagnoses (depression/anxiety disorders), and (3) recent psychotropic medication use (within 30 days). Post-EEG quality control led to the exclusion of six patients for EEG drift duration >20% (defined as sustained slow-wave artifacts >100 μV for more than 5-second) and four participants for ≥5 dysfunctional electrodes (defined as impedance consistently >50 kΩ). The final analytical cohort consisted of 45 participants who were systematically categorized into three age- and sex-matched cohorts. Table 1 presents the demographic and clinical characteristics of the three groups.

Acute tinnitus group (AT): 15 patients with symptom duration <6 months, had a mean age of 39.9 ± 9.0 years and a median tinnitus duration of 4 months (interquartile range: 2-5 months). Chronic tinnitus group (CT): 15 patients with symptom duration ≥6 months, had a mean age of 43.3 ± 9.3 years and a median tinnitus duration of 28 months (interquartile range: 12-60 months). Healthy control group (HC):15 individuals with normal audiometric thresholds, had a mean age of 42.3 ± 14.8 years.

#### Clinical assessments

The baseline clinical profiling involved comprehensive audiometric and psychometric evaluations. Hearing thresholds were measured bilaterally using pure-tone audiometry (Otometrics Astera, USA) across frequencies of 0.25–8 kHz, and tympanometry was performed to confirm normal middle ear function. Tinnitus severity was assessed using two standardized instruments.

(1) Tinnitus Handicap Inventory (THI)^54^: A 25-item questionnaire divided into three subscales, functional (THI-F), emotional (THI-E), and catastrophic (THI-C), with total scores ranging from 0 (no handicap) to 100 (severe disability). The responses were scored as “yes” (4 points), “sometimes” (2 points), or “no” (0 points).
(2) Visual Analog Scale (VAS)^36^: A 10-point Likert scale anchored by cartoon expressions (from “no tinnitus” [0] to “unbearable loudness” [10]) to quantify the perceived tinnitus intensity.

Demographic characteristics, including age, sex, tinnitus laterality (left/right/bilateral), and symptom duration, were systematically recorded. Group-averaged audiograms for the three subsamples are presented in Figure 2, confirming matched hearing thresholds across frequencies.

#### EEG acquisition

High-density resting-state EEG recordings were acquired in a controlled electrophysiological environment to minimize external noise interference. Participants were seated in a sound-attenuated, electromagnetically shielded Faraday chamber and instructed to remain relaxed with their eyes closed during the 10-min session. A 64-channel Neuroscan SynAmps2 system (Compumedics, Australia) was used with electrodes arranged according to the extended 10-20 International System. Electrode-scalp impedance was maintained below 10 kΩ using conductive gel (Quick-Gel), and vigilance was monitored through real-time electromyography (EMG) and electrooculography (EOG) to ensure patients were in an awake motion-free state. Signals were digitized at 1,000 Hz, with a 1–45 Hz bandpass filter and 50 Hz notch filter applied to eliminate line noise. Linked mastoids (M1/M2) served as the reference, and raw data were stored using Curry 8 software (Compumedics, Australia) for subsequent processing.

Before recording began, each participant was given standardised verbal instructions: to keep their eyes closed, remain awake and physically still, relax both body and mind without engaging in any specific mental activity, and not to deliberately focus on or suppress the tinnitus. The experimenter continuously monitored real-time electromyography (EMG) and electrooculography (EOG) to verify that the participants remained awake and minimised physical movement. Should any signs of drowsiness appear, a brief verbal reminder was given.

#### EEG preprocessing

Preprocessing of raw EEG data was processed using MATLAB (version R2022b; MathWorks, Natick, MA, USA) with the EEGLAB Toolbox^55^ v2024.1.0, following a semi-automated workflow.

The steps are briefly described as follows: (1)Data import and filtering: Raw EEG data were imported, and a 50 Hz notch filter was applied to remove industrial frequency interference. Finally, a bandpass filter from 0.5 to 45 Hz was applied to the signal. (2)Re-referencing and down-sampling: Non-cephalic electrodes (e.g., EKG) were removed. The data were re-referenced to the average of all the cerebral electrodes, and the sampling rate was adjusted to 500 Hz. (3)Generation of power spectral density (PSD): PSD plots for each channel were generated and inspected, and data from channels with abnormally high energy were excluded. (4)Channel interpolation: Data were visually inspected to remove large drifts caused by electrode signal instability. Individual bad channels (exhibiting flatline, excessive noise, or high impedance) were marked for spherical interpolation; however, recordings containing ≥5 such channels were excluded rather than interpolated. The aforementioned determinations were independently conducted by two trained researchers. (5)Artifact correction: To further reduce the influence of volume conduction effects in scalp signals, independent component analysis (ICA) was applied to compute temporally independent components. Artifactual components (e.g., ocular, muscular, cardiac, and electrode noise) were automatically identified and labeled using the ICLabel plugin. Components with a probability ≥ 90% of belonging to any non-brain source category (i.e., “Eye,” “Muscle,” “Heart,” “Line Noise,” or “Channel Noise”) were excluded from subsequent analysis. Independent components classified by ICLabel as non-brain components with probabilities between 70% and 90% underwent additional manual inspection. Specifically, (a) scalp topographies were examined to identify typical brain-related spatial patterns and to exclude characteristic artifact patterns, such as frontal ocular artifacts, focal noisy electrodes, and temporalis or cervical muscle activity; (b) power spectral density profiles were inspected for abnormal 50 Hz line-noise peaks and excessive broadband high-frequency activity (>30 Hz), which are commonly indicative of residual muscle contamination. The average number of retained ICA components was approximately 47 in the AT group (range: 41-53), approximately 48 in the CT group (range: 41-54), and approximately 47 in the HC group (range: 44-52). (6) Epoch segmentation and validation: Three non-overlapping, artifact-free 10-second segments were selected for each participant, yielding a total of 30 seconds of high-quality data per subject for microstate analysis. In accordance with established protocols,^19^^,^^56^ global field power (GFP) peaks were identified from these continuous segments, providing a sufficient number of stable topographies for robust clustering. All data were visually inspected for artifacts (e.g., muscle bursts and eyeblinks) and abnormal PSD patterns.

#### Dynamic resting-state functional network calculations

The full pipeline discussed in this section is summarized in Figure 1 and was divided into two steps.

##### EEG segmentation based on microstates

EEG recordings were clustered and segmented into distinct microstates using the EEGLAB Microstate Analysis Toolbox.^57^ This process includes the following steps.

(a) Preprocessing enhancement: A secondary bandpass filter (1–40 Hz, zero-phase finite impulse response [FIR]) was applied to reduce high-frequency noise and slow drifts before microstate extraction to obtain the frequency band required for EEG microstate analysis.^56^
(b) Global field power (GFP) Calculation: GFP, representing the instantaneous global electric field strength, was computed as the spatial standard deviation across the channels. Because the local maximum position of GFP is considered to have the best signal-to-noise ratio and corresponds to stable topographic maps,^17^^,^^58^ the time series of the GFP maximum value were further extracted (with 1000 random points for each subject). The GFP was then computed as follows:

(Equation 1) $$\begin{array}{c} GFP\left(t\right)=\sqrt{\frac{1}{K}\sum_{i=1}^{K}{\left(V_{i}\left(t\right)-V_{mean}\left(t\right)\right)}^{2}} \end{array}$$

Where *K* denotes the number of electrodes in the EEG data, *V*_*i*_(*t*) is the instantaneous potential of the *i*-th electrode at time point *t*, and *V*_*mean*_(*t*) is the average of all lead transient potentials at time point *t*.

(c) Clustering and classification: A modified K-means clustering algorithm called ‘*Modkmean*’ was used to construct a ‘*microstate model.*’ Four brain topographic maps were randomly selected from the GFP maximum time series as the ‘*initial cluster center.*’ These maps were then compared with the remaining topographic maps; the most similar maps were marked, and ‘*new cluster centers*’ were calculated from them. This step was repeated a hundred times until the percentage of EEG signals represented by the cluster centers no longer increased, at which point the four cluster centers obtained represented the ‘*microstate model*’ of the study participant. Finally, EEG microstates A, B, C, and D were calculated from the group-level ‘*microstate model*’ of all participants and validated against prior literature for spatial similarity.^22^^,^^59^ The quality of clustering was evaluated via global explained variance (GEV, the proportion of total EEG variance explained by the four canonical microstate templates, expressed as a percentage), and individual maps were labeled based on prototypical features^60^:
A: Right anterior-left posterior dipole
B: Left anterior-right posterior dipole
C: Anterior-posterior orientation
D: Fronto-central extreme location

##### EEG functional network calculations

Imaginary phase-locked values (iPLVs) were used as a functional connectivity metric. This measure is particularly well-suited to the microstate concept, given that phase-locked neural activity can generate stable topographies in scalp EEG recordings. Furthermore, iPLV is highly sensitive to interactions with non-zero phase lags while being robust to instantaneous self-interactions that may arise from volume conduction. Thus, iPLV was suitable for our analysis. For each participant, EEG functional network calculations were divided into the following two steps:

(a) Calculation of the instantaneous phase of EEG recordings across six frequency bands. First, a FIR filter was applied to split EEG recordings into six frequency bands: δ (1–4 Hz), θ (4–8 Hz), α-low (8–10 Hz), α-high (10–13 Hz), β (13–30 Hz), and γ (30–40 Hz). Then, the instantaneous phase in the EEG recordings was calculated via Hilbert transformation.
(b) Construction of functional networks corresponding to different microstates and frequency bands based on iPLV: In our analysis, iPLV was computed directly between sensor pairs, without performing source reconstruction, thereby preserving the original sensor-space signals while mitigating volume conduction artifacts. For each frequency band, the iPLV of a given microstate was defined as the mean iPLV of all segments corresponding to that microstate. The iPLV for each EEG segment was defined as follows:

(Equation 2) $$\begin{array}{c} iPLV=\left|\left\langle im\left(e^{i\Delta \varphi \left(t\right)}\right)\right\rangle \right| \end{array}$$

Where Δ*φ*(*t*) = *φ*_*y*_(*t*)-*φ*_*x*_(*t*), *φ*_*x*_(*t*), and *φ*_*y*_(*t*) are two phase signals, and ⟨.⟩ is the averaging operator. In a discrete case, *t* represents the sampling point within a single segment.

#### Dynamic analysis

This section has two parts. First, the temporal properties of the microstate sequences were calculated for temporal dynamic analysis. Second, subnetwork topologies were computed based on the graph theory.

##### Temporal properties

Five temporal properties were selected. Microstate time series contain numerous parameters with potential neurophysiological significance. In general, for each microstate class, the main EEG microstate metrics used to describe changes in the brain state are as follows^19^^,^^61^^,^^62^:

(a) GEV: The proportion of total variance explained by the microstate,
(b) Duration: The average length of time the microstate remains stable whenever it appears,
(c) Occurrence: The average number of times per second the microstate becomes dominant during the recording period,
(d) Coverage: The proportion of total recording time occupied by the microstate.
(e) Transition probabilities (TP): Reflect switching between different brain networks in the brain (e.g., B to D).

##### Subnetwork topologies

We quantified each functional subnetwork of the DFNs using global efficiency (GE), a metric derived from the graph theory. This metric was calculated using the Brain Connectivity Toolbox.^63^ The GE can then be computed as:

(Equation 3) $$\begin{array}{c} E_{glob}=\frac{1}{N*\left(N-1\right)}\sum_{i\neq j\in G}{\left(d_{ij}\right)}^{-1} \end{array}$$

Where *G* is a graph composed of *N* nodes, and *d*_*ij*_ is the shortest weighted path length between nodes *i* and *j*.

To validate the representativeness of this approach, we conducted a test-retest consistency analysis using an independent set of three non-overlapping 10-second segments randomly selected from each participant's 10-minute recording. The selection of three 10-second artifact-free segments follows established protocols in EEG microstate studies.^32^^,^^42^ We repeated the entire microstate analysis pipeline on these alternative segments and compared the two sets of results using two complementary statistical approaches: Pearson correlation coefficients to assess linear associations and intraclass correlation coefficients (ICC) to evaluate absolute agreement, using the ICC(2,1) model (two-way random effects, single measures). The results demonstrated good to excellent stability for the core microstate parameters (see Table S1 for full consistency data).

### Quantification and statistical analysis

Statistical analyses were performed using SPSS 29.0 (IBM Corp., Armonk, NY, USA) and GraphPad Prism 8.0 (GraphPad Software, Inc., La Jolla, CA, USA). Normality was assessed using the Shapiro-Wilk test and Q-Q plots, and homogeneity of variance was assessed using Levene’s test. For normally distributed variables, center and dispersion measures are reported as mean ± standard deviation (SD); for non-normally distributed variables, they are reported as median (first quartile, third quartile). A two-tailed significance threshold of *p* < 0.05 was applied, and Cohen's d was used to quantify effect sizes for significant findings.

Statistical details for each analysis are reported as follows:

(1) Demographic and clinical characteristics (Table 1): Sex and tinnitus laterality distributions were assessed using the Chi-square test. Age and bilateral PTA were compared across the three groups using one-way ANOVA followed by Bonferroni post-hoc tests. Independent samples t-tests were used for two-group comparisons of tinnitus-ear PTA, THI total score, and THI subscales (functional, emotional, and catastrophic) between the AT and CT groups. Mann–Whitney U tests were used for two-group comparisons of tinnitus duration and VAS between the AT and CT groups.
(2) Microstate temporal parameters and global explained variance (Figure 4 and Table 2): Group differences in GEV, microstate duration, occurrence, and coverage were assessed using one-way ANOVA followed by Bonferroni-corrected pairwise post-hoc comparisons. Effect sizes were quantified using Cohen's d.
(3) Microstate transition probabilities (Figure 5): Group differences in transition probabilities were assessed using one-way ANOVA followed by Bonferroni-corrected pairwise post-hoc comparisons.
(4) Static and dynamic functional network global efficiency (Figure 6 and Table 3): Group differences in global efficiency across six frequency bands and four microstate classes were assessed using one-way ANOVA followed by Bonferroni-corrected pairwise post-hoc comparisons. Post-hoc P values and Cohen's d are detailed in Table 3.
(5) Test-retest consistency analysis (Table S1): Pearson correlation coefficients and intraclass correlation coefficients (ICC) were computed to evaluate the stability of microstate parameters and functional connectivity metrics between the original 30-second epochs and alternative 10-second segments. ICC values were computed using the ICC(2,1) model (two-way random effects, single measures).

No formal sample size estimation (power analysis) was performed for this exploratory study; the sample size was determined based on practical recruitment considerations and was comparable to previous EEG microstate studies in tinnitus.

Participants were allocated to groups based on tinnitus duration (AT: <6 months; CT: ≥6 months) with age- and sex-matched assignment; no additional randomization or stratification procedures were applied as this was an observational case-control study.

Predefined inclusion and exclusion criteria (see experimental model and study participant details and method details) were applied uniformly to all participants, and data were checked to ensure that they met the assumptions of each statistical test (normality via Shapiro-Wilk test and Q-Q plots; homogeneity of variance via Levene’s test) prior to analysis. No data or subjects were excluded from the analyses after statistical procedures were initiated.
