# Supplementary material for: EEG microstates and dynamic functional connectivity reveal stage-specific brain networks in subjective tinnitus
Source: iScience. 2026 Jul 30;29(8):116995. doi: 10.1016/j.isci.2026.116995 (PMC13452246; doi:10.1016/j.isci.2026.116995)
Supplement: Supplementary file 1 [file mmc1.pdf]

iScience, Volume *29*

Supplemental information

EEG microstates and dynamic functional connectivity reveal stage-specific brain networks in subjective tinnitus

Meng-Fang Gong, Sheng-Yu Tao, Bin Dai, Zhong-Ling Ding, Qian He, Ya-Kang Dai, Ji-Sheng Liu, and Duo-Duo Tao

Table S1. Consistency between the original and alternative segments for microstate and connectivity parameters.

Parameter Category			Pearson r	ICC
Global Explained Variance (GEV)			0.899	0.895
Microstate Duration		A	0.760	0.320
		B	0.930	0.682
		C	0.465	0.260
		D	0.897	0.480
Microstate Coverage		A	0.908	0.905
		B	0.934	0.901
		C	0.834	0.828
		D	0.939	0.900
Microstate Occurrence		A	0.881	0.589
		B	0.819	0.384
		C	0.788	0.345
		D	0.845	0.594
Transition Probabilities		A to B	0.771	0.746
		A to C	0.746	0.735
		A to D	0.806	0.743
		B to A	0.782	0.781
		B to C	0.799	0.796
		B to D	0.852	0.858
		C to A	0.839	0.835
		C to B	0.847	0.838
		C to D	0.825	0.802
		D to A	0.682	0.692
		D to B	0.661	0.665
		D to C	0.665	0.659
SFN		δ	0.824	0.796
		θ	0.912	0.899
		α-low	0.92	0.923
		α-high	0.937	0.939
		β	0.903	0.907
		γ	0.836	0.826
DFN	δ	A	0.606	0.615
		B	0.798	0.739
		C	0.765	0.768
		D	0.678	0.686
	θ	A	0.705	0.685
		B	0.786	0.776
		C	0.699	0.703
		D	0.842	0.848

α-low	A	0.769	0.777
	B	0.873	0.876
	C	0.927	0.929
	D	0.916	0.919
α-high	A	0.871	0.865
	B	0.834	0.836
	C	0.909	0.91
	D	0.907	0.909
β	A	0.697	0.691
	B	0.534	0.509
	C	0.768	0.728
	D	0.798	0.804
γ	A	0.543	0.542
	B	0.759	0.738
	C	0.813	0.802
	D	0.68	0.684

Note: Pearson correlation coefficients and intraclass correlation coefficients (ICC) comparing the original 30-second analysis epochs with an independent set of three alternative 10-second segments. ICC values were computed using the ICC(2,1) model (two-way random effects, single measures). SFN, static functional network; DFN, dynamic functional network; GEV, global explained variance.
